# Coinfections and superinfections in critically ill COVID-19 patients in Ecuador: a cross-sectional study of bacterial and viral pathogens

**DOI:** 10.3389/fpubh.2025.1664521

**Published:** 2025-12-04

**Authors:** Diana Morales-Jadán, Claire Muslin, Carolina Viteri-Dávila, Jorge Luis Vélez-Páez, Estefanía Belen Irigoyen-Mogro, Nikolaos C. Kyriakidis, Miguel Angel Garcia-Bereguiain, Ismar A. Rivera-Olivero

**Affiliations:** 1 One Health Research Group, Universidad de Las Américas, Quito, Ecuador; 2 Facultad de Ciencias Médicas, Universidad Central del Ecuador, Quito, Ecuador; 3 Hospital Pablo Arturo Suarez, Unidad de Terapia Intensiva, Centro de Investigación Clínica, Quito, Ecuador; 4 Dirección General de Investigación y Vinculación, Universidad de Las Américas, Quito, Ecuador; 5 Center for Hematology and Regenerative Medicine, Department of Medicine Hudddinge, Karolinska Institute, Stockholm, Sweden; 6 One Health Research Group, Facultad de Medicina, Universidad de Las Américas, Quito, Ecuador

**Keywords:** superinfection, ICU, coinfection, COVID-19, *S. pneumoniae*

## Abstract

**Introduction:**

The COVID-19 pandemic has deeply affected Latin America and Ecuador. Coinfections and superinfections increase the risk of morbidity and mortality in COVID-19 patients. This study examined co-infections and superinfections in critically ill COVID-19 patients admitted to the ICU of a tertiary hospital in Ecuador.

**Methods:**

A cross-sectional study was conducted from February to August 2021, including patients with a confirmed SARS-CoV-2 infection. Demographic data, clinical characteristics, and microbiological findings were analyzed to evaluate the presence of coinfections and superinfections.

**Results:**

A total of 24 patients were included, of whom 70.83% (17/24) experienced either coinfection or superinfection. Community-acquired coinfections were identified in 12.5% (3/24) of patients, whereas hospital-acquired superinfections were detected in 58.3% (14/24). The most frequently isolated pathogens were *Klebsiella pneumoniae*, *Staphylococcus aureus*, and *Enterococcus faecalis*. Molecular testing revealed Streptococcus pneumoniae was the most prevalent organism. Bloodstream infections were the most common superinfections, with an attack rate of 92.8% (13/14). The median time from hospital admission to superinfection diagnosis was 5 days. The study also found that 33% (8/24) of patients died, all of whom were men; 62% (5/8) of the patients who died have superinfection. However, infections were not identified as independent predictors of death, given the small cohort size (*n* = 24) and descriptive statistical design.

**Discussion:**

These findings underscore the importance of robust monitoring of co-infections and superinfections in critically ill COVID-19 patients, especially in resource-limited settings. The high prevalence of these infections highlights the need for continued investment in microbiological surveillance, rapid diagnostics, and antimicrobial stewardship programs to mitigate long-term consequences and address the increasing threat of antimicrobial resistance.

## 1 Introduction

Since its emergence in December 2019, COVID-19 remains a significant threat to global health. It is characterized primarily by respiratory symptoms, common clinical manifestations such as fever (81.2%), and respiratory signs and symptoms such as cough and dyspnea (1, 2).

On June 29, 2025, more than 778 million COVID-19-confirmed cases were reported (3), with nearly 7 million reported deaths worldwide, extremely challenging health systems, and vulnerabilities in surveillance systems. Although the pandemic has had global repercussions, Latin America has been one of the most profoundly affected regions, accounting for approximately 25% of global infections (4), partly because of structural inequities, a lack of diagnostic capacity, and irregular access to care (5).

The case fatality rate (CFR) of COVID-19 has been estimated at 2.7%, although it varies substantially among patients with underlying comorbidities (3). More severe outcomes requiring mechanical ventilation and intensive care are more frequent among older individuals and those with preexisting conditions (6–11).

The World Health Organization (WHO) estimates that global excess mortality attributable to the COVID-19 pandemic reached 14.83 million deaths from January 2020 to December 2021, 2.74 times higher than the 5.42 million officially reported COVID-19 deaths for the same period (12).

In Ecuador, the first confirmed case was reported on February 29, 2020 (13), and analyses of excess mortality showed that official COVID-19 death counts underestimated the true pandemic impact, the country has the second highest P-score among the countries analyzed, with a 51% increase in deaths over the pandemic period relative to expected levels. This places Ecuador second behind Peru, which had the highest P-score of 97% (12).

Between 2020 and 2024, four countries from Latin America and the Caribbean, Peru, Mexico, Brazil, and Chile were among the 20 countries with the highest COVID-19-related mortality (14). These estimates highlight the substantial impact of the pandemic, extending beyond officially reported COVID-19 fatalities, and capturing both the direct and indirect effects of the pandemic on global mortality.

Coinfections and superinfections increase the risk of morbidity and mortality in COVID-19 patients. Coinfections are defined as the detection of additional pathogens in a patient with an infection within 48 h of hospital admission (15, 16), whereas superinfections develop 48 h after initial infection and hospital admission (17). In critically ill COVID-19 patients, bacterial coinfections may involve both community-acquired pathogens, such as *Streptococcus pneumoniae, Haemophilus influenzae*, and *Staphylococcus aureus*, and hospital-acquired, multidrug-resistant organisms, as well as fungal pathogens (18).

Furthermore, other respiratory viruses, such as influenza, parainfluenza, and respiratory syncytial virus (RSV), present with signs and symptoms similar to those of COVID-19, further complicating the differential diagnosis (2, 19).

Viral respiratory co-infections, including influenza, rhinovirus, respiratory syncytial virus (RSV), and parainfluenza virus, are well recognized as modifiers of disease severity (16, 20). Studies have indicated that respiratory viral pathogens may compete, potentially through mechanisms involving immune-mediated interference, resulting in the downregulation of some viruses during the peak of another virus, a phenomenon that has been consistently observed and described for decades (21, 22).

Among bacterial pathogens, *Streptococcus pneumoniae* remains a leading cause of community-acquired pneumonia globally and in Latin America, where heterogeneity in pneumococcal conjugate vaccine (PCV) coverage persists (19, 23). Viral infections can disrupt mucociliary clearance, alter host immune responses, and facilitate bacterial adherence, thereby increasing the susceptibility to pneumococcal superinfection (21, 24). The clinical relevance of detecting *S. pneumoniae* coinfection lies in its implications for empirical antibiotic therapy, antimicrobial stewardship, and pneumococcal vaccination strategies, especially in resource-limited settings where diagnostic infrastructure is still developing (15, 17).

Several studies have reported different coinfection rates. For example, approximately 5.8% of the COVID-19-positive patients in Wuhan were simultaneously infected with other respiratory viruses (25). In contrast, in Northern California, 20.7% of confirmed COVID-19 cases exhibited co-infection with at least one additional pathogen, most frequently respiratory viruses (9), with influenza being a virus of significant concern (19, 26).

Studies from Latin America reported that bacterial coinfections at hospital presentations in COVID-19 patients were uncommon, while secondary bacterial superinfections were more frequent. In Peru, a prospective study using molecular panels (BioFire FilmArray® Pneumonia Plus) detected bacterial respiratory pathogens in 38 of 93 patients (40.9%) upon hospital admission (27) Whereas in Colombia, and a multicenter study in Medellín reported a 49.6% prevalence of bacterial superinfection (198/399) among hospitalized COVID-19 patients, with *Klebsiella* spp. and *Staphylococcus aureus* being the most common isolates (28). Few cases of superinfection in intensive care unit (ICU) patients with COVID-19 have been reported in Ecuador (29, 30).

Molecular diagnostics has transformed respiratory pathogen detection by simultaneously offering rapid and sensitive identification of multiple targets. However, molecular positivity alone does not confirm active infection, as nucleic acids may be derived from colonizing or non-viable organisms (31, 32). Quantitative or semi-quantitative interpretation of molecular results through cycle threshold (Ct) values or pathogen load can help differentiate colonization from infection. However, clinical correlation remains indispensable (33, 34).

Therefore, molecular assays should be viewed as valuable diagnostic tools that complement, rather than replace, traditional microbiological methods and clinical assessment. Integration of molecular findings with radiological evidence, biomarker data (e.g., procalcitonin), and culture or antigen results provides a more accurate framework for distinguishing true infection from colonization and for guiding antimicrobial therapy (8).

Coinfections and superinfections are considered contributing to the increased severity and fatality of SARS-CoV-2 infection in low-resource countries. Therefore, the timely detection of these infections is vital for initiating appropriate antimicrobial interventions and preventing clinical deterioration.

By combining molecular and conventional microbiological methods, we sought to improve our understanding of viral and bacterial coinfection and superinfection dynamics in an underrepresented Latin American region to generate evidence that can inform diagnostic and therapeutic strategies in similar healthcare settings.

This study aimed to determine the prevalence of viral and bacterial coinfections and superinfection among critically ill patients with SARS-CoV-2 admitted to the intensive care unit of the General Pablo Arturo Suárez Provincial Hospital in Ecuador.

## 2 Materials and methods

### 2.1 Study design and participants

This cross-sectional study was conducted in Quito-Ecuador between February and August 2021, at the Hospital Provincial General Pablo Arturo Suárez. This study included all patients admitted to the intensive care unit (ICU) who met the clinical diagnostic criteria for COVID-19 and COVID-19 pneumonia. Inclusion required compatible clinical and radiological findings, along with a confirmed positive result on real-time reverse transcription polymerase chain reaction (RT-PCR) assay for SARS-CoV-2 from a respiratory specimen (nasopharyngeal swab, tracheal aspirate, and bronchoalveolar lavage). The WHO COVID-19 Disease Severity Classification System was used to define severe and critical COVID-19 (35). Patients with acute respiratory distress syndrome were classified according to the Berlin definition (36). None of the eligible patients was excluded from the study.

Demographic data (age, sex, and occupation), clinical characteristics (comorbidities, laboratory test results at ICU admission, microbiological results, and date of admission), treatment, and outcomes were collected directly from the health records. Screening of bacterial pathogens in the blood, urine, tracheal aspirate, sputum, and other samples was performed by culture using standard microbiological methods at the hospital’s clinical laboratory as part of the standard of care based on attending physicians’ orders.

Detection of *S. pneumoniae* and respiratory viruses, including SARS-CoV-2, was performed in the laboratory of Universidad de Las Americas using tracheal aspirate samples collected 1–5 times during each patient’s ICU hospitalization, until discharge or death.

### 2.2 Definitions

*Bloodstream infection*: defined as the growth of a recognized pathogen (non-commensal) in ≥1 blood cultures. For skin commensals such as coagulase-negative *Staphylococcus*, ≥2 positive cultures with clinical relevance (validated by a study physician) were required.

*Lower respiratory tract infection*: Bacterial growth in quantitative lower respiratory cultures above the diagnostic threshold, fungal growth, or a positive polymerase chain reaction (PCR) result from respiratory specimens with compatible clinical findings.

*Urinary tract infection*: Defined as a positive urine culture in a symptomatic patient.

#### 2.2.1 Definition of coinfection and superinfection

Coinfections and superinfections were classified based on timing, microbiological criteria, and clinical correlation. Coinfection was defined as the detection of a non–SARS-CoV-2 pathogen by culture or molecular assays within the first 48 h of hospital admission. In contrast, superinfection (secondary infection) was defined as the detection of a new pathogen 48 h after hospital admission, accompanied by new or worsening clinical or radiological findings compatible with infection.

When both polymerase chain reaction (PCR) and culture results were available, culture-positive findings from sterile sites (e.g., blood or pleural fluid) or quantitative lower respiratory cultures exceeding accepted diagnostic thresholds (bronchoalveolar lavage ≥10^4^ CFU/mL, protected specimen brush ≥10^3^ CFU/mL, or endotracheal aspirate ≥10^5^ CFU/mL) were considered definitive evidence of infection. PCR-positive but culture-negative results were regarded as probable infections only when obtained from lower respiratory specimens showing a high pathogen load and consistent clinical manifestations. Conversely, PCR detection of potential colonizers in upper airway samples without supportive clinical, radiological, or quantitative evidence was not classified as an infection.

All diagnostic definitions and thresholds were adapted from the Infectious Diseases Society of America/American Thoracic Society (IDSA/ATS) guidelines for hospital-acquired and ventilator-associated pneumonia (37) and from previous studies assessing co-infections and superinfections in COVID-19 patients (38–41).

### 2.3 Procedures

Tracheal aspirate samples were divided into two aliquots. One aliquot was subjected to enzymatic digestion before DNA extraction to detect S. *pneumoniae*. The second aliquot was used for direct extraction of nucleic acids for the detection of SARS-CoV-2 and other respiratory viruses.

### 2.4 Digestion and nucleic acid extraction

To disrupt the *S. pneumoniae* capsule, tracheal aspirate samples were subjected to an enzymatic digestion protocol for 8 h at 36 °C, prior to nucleic acid extraction. A total of 100 μL of TE buffer (10 mM Tris–HCl, 1 mM EDTA, pH 8.0) containing 0.04 g/mL lysozyme (Sigma) and 75 U/mL mutanolysin (Sigma) was mixed with 200 μL of sample material (42). Viral DNA and RNA were co-extracted using a spin-column-based extraction kit (Biocomma Limited, Guangdong, China).

### 2.5 Detection of SARS-CoV-2 by RT-qPCR

The presence of SARS-CoV-2 in tracheal aspirate samples was determined using the CDC 2019-Novel Coronavirus (2019-nCoV) RT-PCR Diagnostic Panel. In summary, the assay developed by the CDC and authorized by the FDA under Emergency Use Authorization (EUA) utilizes the 2019-nCoV CDC kit (IDT, USA), which targets the N1 and N2 regions of the virus for detection, along with the RNase P gene as an internal control for RNA extraction quality. Negative controls consisting of TE buffer (pH 8) were included in each run to detect potential carryover contamination and guarantee that only true positives were reported. A concentration of 200.000 genome equivalents/mL of the 2019-nCoV N positive control (IDT, USA) was used for viral load calculation (43–45).

### 2.6 Detection of *S. pneumoniae* by qPCR

Detection of *S. pneumoniae* was detected using previously reported primers and probes (46). Primers and probes targeting *LytA* were designed and validated by the Center for Disease Control and Prevention (CDC). The sequences used were as follows: *LytA* forward primer: ACGCAATCTAGCAGATGAAGCA; *LytA* reverse primer: TCGTGCGTTTTAATTCCAGCT; and probe: 5’-FAM-TGCCGAAAACGC’T’TGATACAGGGAG-3’-SpC, where ‘T’ is labeled with BHQ1. The reference accession number is EA005672. Additionally, CDC-approved multiplex real-time PCR assays were employed to detect 21 pneumococcal serotypes or serogroups across seven PCR reactions in *LytA-*positive samples (47).

### 2.7 Multiplex RT-qPCR detection of 12 respiratory viruses

RNA was extracted from tracheal aspirate samples and reverse-transcribed into complementary DNA (cDNA) using Invitrogen™ SuperScript™ II Reverse Transcriptase (200 U/mL), along with 10X RT Buffer, 10X random primers, 10 mM dNTPs, RNAseOUT™ (40 U/mL), and MultiScribe reverse transcriptase.

Four real-time multiplex PCR assays were then performed to identify the presence of 12 different respiratory viruses, including influenza A virus, influenza B virus, rhinovirus, adenovirus, respiratory syncytial virus (RSV) A/B, Human metapneumovirus (HMPV), parainfluenza virus types 1, 2, and 3, and HCoV types NL63, 229E, and HKU1, as previously described by Morales-Jadan et al. (48).

### 2.8 Statistical analysis

Statistical analyses were performed using SPSS version 28.0 for Windows (IBM Corp., Armonk, NY, USA). Continuous variables were summarized as medians and interquartile ranges (IQRs), while categorical variables were expressed as counts and percentages. Demographic and clinical characteristics including age, sex, occupation, symptoms, and comorbidities were examined in the study cohort. The number of SARS-CoV-2 positive and negative individuals was recorded to calculate the attack rate. Thirteen respiratory pathogens were analyzed in SARS-CoV-2–positive patients. Comparisons between groups were performed using the chi-square test or Fisher’s exact test when appropriate, with a *p*-value < 0.05, indicating statistical significance. All analyses were descriptive and exploratory, and no multivariable adjustments for confounders were made because of the small sample size. Mortality analyses in relation to co-infections and superinfections were also explored to generate hypotheses rather than to establish statistical associations.

### 2.9 Ethics statement

The research protocol was reviewed and approved by the Ethics Committee of the National Health Intelligence Directorate of the Ministry of Public Health (Dirección Nacional de Inteligencia de la Salud, Ministerio de Salud Pública, Ecuador) approval code N° 008-2020. Written informed consent was obtained from all participants or their authorized representatives.

## 3 Results

### 3.1 Patient characteristics

During the study period, 24 patients were admitted to the ICU (intensive care unit) with confirmed community-acquired pneumonia due to SARS-CoV-2 infection were admitted to the intensive care unit.

All patients (100%) tested positive for COVID-19 by RT-PCR, and 83.3% (*n* = 20) fulfilled the criteria for severe acute respiratory distress syndrome (ARDS). Most patients were male (87.5%, *n* = 21). Patient ages ranged from 22 to 75 years, with a mean age of 48 years.

Comorbidities were present in 37.5% (*n* = 9) of patients, with obesity being the most frequently reported condition, followed by arterial hypertension. Summary of sociodemographic characteristics, occupational distribution, pre-existing comorbidities, and baseline inflammatory and pro-thrombotic parameters (with 95% confidence intervals) measured at ICU admission in patients with confirmed COVID-19 are in Table 1.

**Table 1:** Baseline sociodemographic features, occupation types, comorbidity profiles, and inflammatory and coagulation biomarkers at the time of ICU admission in adults with confirmed SARS-CoV-2 infection.

Patient	Data	*N* = 24
Characteristic	Male, *n* (%)	21 (87.5%)
	Female, *n* (%)	3 (12.5)%
	Age (years), mean	22–75, 48
Occupation	Agricultural work	1 (4.2%)
	Domestic work	2 (8.3%)
	Commerce	5 (20.8%)
	Trades	3 (12.5%)
	Office/administrative work	1 (4.2%)
	Transportation	7 (29.2%)
	Unemployed	5 (20.8%)
Comorbidities	Obesity	4 (16.6%)
	Arterial hypertension	3 (12.5%)
	Rheumatoid arthritis	1 (4.1%)
	Pancreatitis	1 (4.1%)
	Diabetes mellitus	1 (4.1%)
	Hematologic disease	1 (4.1%)
Baseline Inflammatory and pro-thrombotic markers on admission to ICU (95% CI)	D-dimer (ng/ml)	990.5 (691.3–1289.6)
	Ferritin (ng/ml)	1031.7 (735.4–1328.1)
	Lactate dehydrogenase (U/L)	1121.5 (671.5–1571.5)
	Leucocyte count (x10^3^ cells/mm^3^)	10.5 (8.87–12.2)

### 3.2 Clinical presentation

All patients were diagnosed with SARS-CoV-2 infection using PCR before or at the time of hospital admission. Common symptoms at disease onset included dyspnea (79.16%), fever (62.5%), cough (58.3%), and asthenia (58.3%). Frequency and percentage of major symptom categories including respiratory, gastrointestinal, musculoskeletal, sensorineural, cardiac, neurological, and systemic manifestations observed at the time of ICU admission in patients with confirmed COVID-19 (*N* = 24) are presented in Table 2.

**Table 2:** Respiratory, gastrointestinal, musculoskeletal, sensorineural, cardiac, neurological, and systemic symptoms reported at ICU admission in COVID-19 patients.

Symptoms		Patients *N* = 24
Respiratory	Cough	14 (58.3%)
	Dyspnea	19 (79.2%)
	Nasal congestion	4 (16.6%)
	Tachypnea	5 (20.8%)
Gastrointestinal	Vomiting	1 (4.1%)
Musculoskeletal	Myalgia	2 (8.3%)
	Arthralgia	4 (16.6%)
	Chest pain	1 (4.1%)
Sensorineural	Ageusia	1 (4.1%)
	Anosmia	2 (8.3%)
Cardiac	Tachycardia	4 (16.6%)
Neurological	Seizures	1 (4.1%)
	Headache	11 (45.8%)
Systemic	Fever	15 (62.5%)
	Chills	2 (8.3%)
	Asthenia	14 (58.3%)

### 3.3 Coinfections and superinfections

Among the 24 patients, 17 (70.83%) had either a coinfection or superinfection.

Community-acquired coinfections were identified in three patients (12.5%), including two viral co-infections with influenza A, one with human coronavirus NL63 (HCoV-NL63), and one bacterial co-infection with *S. pneumoniae*. All coinfections were detected in tracheal aspirate specimens collected upon admission.

**Figure 1:**
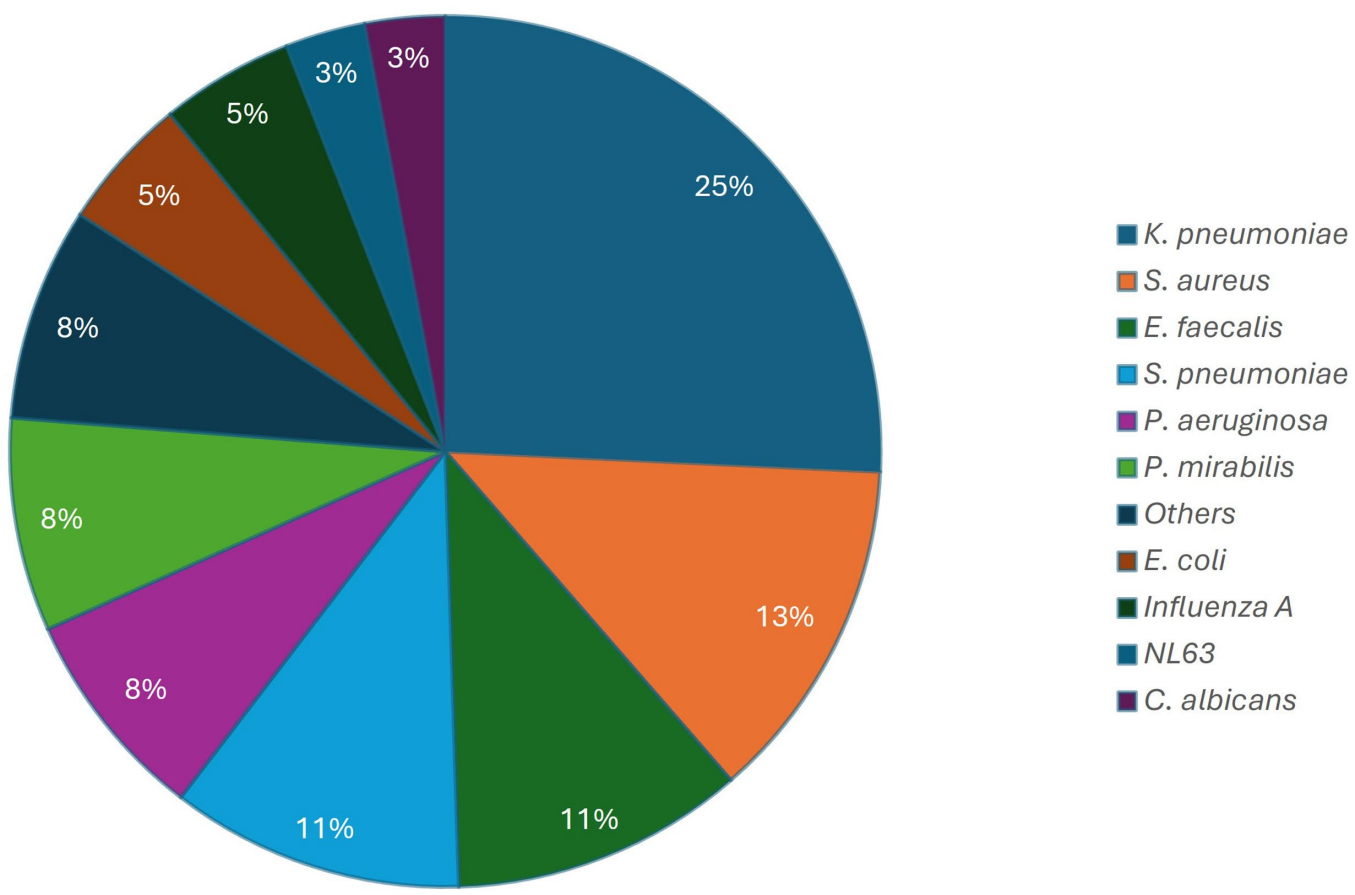
Distribution of microorganisms isolated from ICU patients with COVID-19.

Among the study population, hospital-acquired superinfections were identified in 14 patients (58.3%), including a total of 38 distinct infection episodes, of which 34 were bacterial, three viral, and one fungal. The most frequently isolated pathogens were *Klebsiella pneumoniae* (26%, 10/38), *Staphylococcus aureus* (13%, 5/38), *Enterococcus faecalis* and *S. pneumoniae* (11%, 4/38), *Pseudomonas aeruginosa, Proteus mirabilis* and other bacilli (8%, 3/38), and *Escherichia coli* (5%, 2/8). There were two cases of Influenza A virus infection, one case of NL63, and one case of *Candida albicans* infection (Figure 1).

Among the four patients who tested positive for *S. pneumoniae*, two were identified as serotype 19A, one as serotype 6C/6D, and one as serotype 1.

The most common hospital-acquired superinfection was bacteremia, detected in 13 of 14 patients (attack rate: 92.8%), occurring between days 5 and 46 of ICU stay, followed by respiratory and urinary infections.

The median time from hospital admission to superinfection diagnosis was 5 days (interquartile range [IQR]: 1–20). No significant association was identified between the duration of stay in the intensive care unit (ICU) and the occurrence of superinfection.

**Figure 2:**
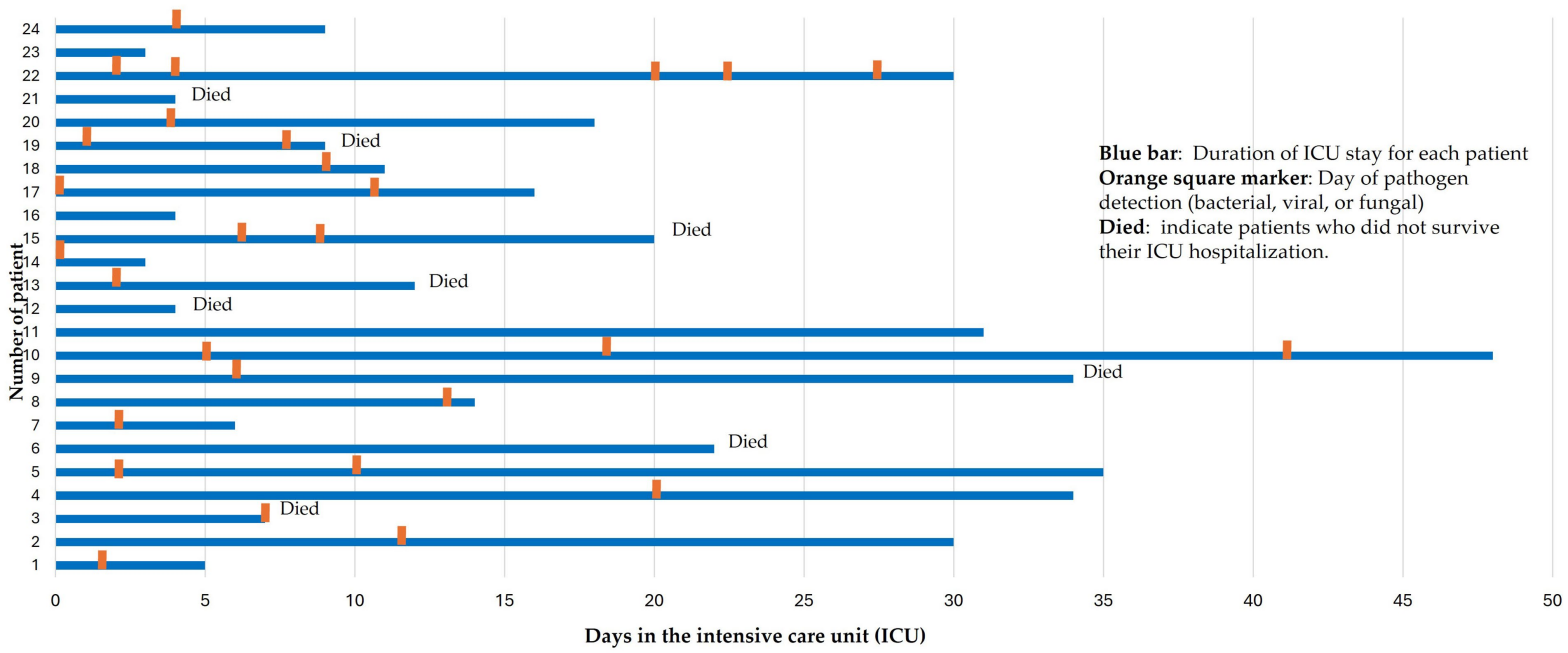
Temporal distribution of pathogen detection during ICU hospitalization among 24 patients with COVID-19.

Figure 2 shows the temporal distribution of pathogen detection among the 24 ICU hospitalized COVID-19 patients. Each horizontal bar represents the duration of ICU stay, whereas orange markers indicate the days when bacterial, viral, or fungal pathogens were detected. This variability may reflect heterogeneity in clinical progression, antimicrobial exposure, and timing of microbiological sampling across patients. Early detection (within the first 48 h) represents co-infections, whereas later detection (after 48 h) corresponds predominantly to superinfections acquired during hospitalization. The figure also shows that several patients with superinfections subsequently died, suggesting a potential association between prolonged ICU stay, the development of infections, and adverse outcomes. However, given the small sample size, these findings should be interpreted as exploratory, rather than inferential.

The median time from hospital admission to ICU admission was 2.5 days (IQR: 1–8 days). The overall median length of UCI stay was 17 days (IQR: 3–46). Most patients (*n* = 23; 95.8%) required intubation, 18 (75%) were placed in the prone position, and four (16.6%) received mechanical ventilation. The Acute Physiology and Chronic Health Disease Classification System II (APACHE II), GLASGOW, and SOFA scores were 14 (range: 5–25), 3 T/15, and 6 (range: 2–13), respectively. Throughout the course of admission, 22 patients (91.6%) received antibiotic therapy during hospitalization.

### 3.4 Mortality and virologic status

The median time to death among the ICU patients was 14 days (IQR: 4–32). Eight patients (33%) died, all of whom were male. Among the deceased, five (62.5%) were aged 48 years or younger and three (37.5%) were aged between 54 and 58 years. Three deceased patients (37.5%) had comorbidities and five (62.5%) (5/8) had superinfections. However, no statistically significant association was identified between the presence of coinfections, superinfections, comorbidities, and fatal outcomes.

At ICU discharge, only five patients tested negative for SARS-CoV-2 in the tracheal aspirate samples. A total of 21 patients (87.5%) remained positive during their ICU stay, while three (12.5%) were SARS-CoV-2-negative upon ICU admission.

Negative SARS-CoV-2 RT-PCR results observed in some ICU patients could reflect the late stage of infection or post-viral phase, rather than the true absence of viral disease, as all patients had previously confirmed COVID-19 diagnosis and compatible imaging findings.

In total, 111 clinical samples were analyzed for microbiological diagnosis (by culture or PCR), comprising (in order of frequency) tracheal aspirates, 61.3% (*n* = 68); blood, 18.8% (*n* = 22); urine, 11.7% (*n* = 13); and other specimen types accounted for the remaining 8.2%.

## 4 Discussion

The COVID-19 pandemic has had a significant impact across Latin America, placing immense strain on healthcare systems already burdened by limited resources and a high prevalence of several comorbidities such as hypertension, diabetes, and respiratory diseases.

This study aimed to assess the incidence and characteristics of coinfections and superinfections in critically ill COVID-19 patients admitted to the ICU of a tertiary hospital in Ecuador.

In our cohort, 70% (17/24) of the patients experienced either a coinfection or superinfection, which was substantially higher than that reported in comparable studies from Guangzhou (2.1%) (49), Seattle (no respiratory virus detected) (50), and France (28%) (51). The elevated rate observed in our study likely reflects both the prolonged ICU stay and the challenges inherent to healthcare delivery in resource-limited settings.

Our cohort showed 12.5% coinfection and 58.3% superinfections. High to systematic reviews have shown that community-onset coinfections are relatively uncommon (3.5%), while ICU-acquired superinfections occur in 14.3% of critically ill COVID-19 patients (39).

The median time to death among the ICU patients was 14 days. Eight patients (33%) died, of whom five (62.5%) had superinfections, suggesting that superinfections may have contributed to poorer outcomes. However, given the small sample size (*n* = 24) and the limited statistical power of our analyses, these findings should be interpreted cautiously and considered exploratory.

The observed temporal pattern is consistent with reports from Brazil, where secondary infections accounted for 42% (89/212), of which 83.14% died (52, 53). In Brazil, 35% of patients hospitalized in ICUs with COVID-19 were co-infected with *Enterobacter* spp., 27% with *Staphylococcus aureus* and 21% with *Klebsiella* spp. The most common coinfections were bloodstream infections, urinary, and respiratory infections (53).

In our study, bloodstream infections were identified in 13 of the 14 patients with superinfections, corresponding to an attack rate of 92.8%, occurring between days 5 and 46 of the ICU stay. This rate notably exceeds the previously reported ICU-acquired infection range of 25–50% during 15–30 days of risk exposure (54).

The high incidence of these infections in our study may reflect a combination of patient- and system-level factors. Critically ill COVID-19 patients in ICUs often require invasive devices, such as central venous catheters, prolonged mechanical ventilation, invasive procedures, and systematic antibiotic use, which predisposes them to secondary bacteremia.

Pandemic-related resource constraints, including shortages of trained ICU personnel, suboptimal nurse-to-patient ratios, and limited availability of infection control supplies, may further increase the risk of healthcare-associated infections (55). Additionally, delays in microbiological diagnosis and restricted access to molecular or rapid identification tools in some hospitals could have contributed to under recognition and inadequate early treatment. These challenges, combined with the regional burden of multidrug-resistant gram-negative bacteria, likely explain the high incidence of bloodstream infections documented in our cohort (38, 56, 57).

Additionally, methodological heterogeneity among studies, such as the timing of sampling, diagnostic modalities (PCR vs. culture), and inclusion of multiple episodes per patient, can account for differences in prevalence. Our inclusion of all microbiologically confirmed infections during prolonged ICU stay led to a higher cumulative detection rate. Our findings reflect the combined impact of clinical, microbiological, and systemic factors shaping infection dynamics in resource-limited hospitals, underscoring the urgent need for strengthened surveillance and infection control measures across the region, particularly in Ecuador.

Microbiological findings revealed that *Klebsiella pneumoniae* (26%) was the most frequently isolated, followed by *Staphylococcus aureus* and *Enterococcus faecalis* (four cases each). However, molecular testing revealed that *Streptococcus pneumoniae* (11%) was the most prevalent organism. These findings are consistent with previous research indicating that *S. aureus, H. influenzae, S. pneumoniae*, and *Enterobacteriaceae* are common in patients with severe SARS-CoV-2 pneumonia (58) and are consistent with reports highlighting the predominance of gram-negative bacteria in ICU-acquired infections (59). While data on adults remain limited, similar coinfections have been documented in pediatric ICU cohorts, particularly involving respiratory syncytial virus, human metapneumovirus (HMPV), and *Mycoplasma pneumoniae* (50, 60). These findings emphasize the importance of screening for viral coinfections upon ICU admission to avoid misdiagnosis and further transmission (60, 61).

Importantly, our study identified that approximately 58% of coinfections were superinfections, that is, they were acquired within 48 h of hospital admission. This aligns with data from a large Colombian cohort, where 84% of all bacterial coinfections occurred in ICU patients, with pneumonia being the most frequently diagnosed infection (41).

ICU admission substantially increases the risk of exposure to invasive interventions and multidrug-resistant pathogens, thereby extending the hospitalization duration and increasing healthcare costs (62, 63). Distinguishing between community- and hospital-acquired infections is critical for implementing appropriate antimicrobial therapies and guiding infection control strategies (64).

Proteins from *S. pneumoniae* and *S. aureus* facilitate influenza virus replication (65, 66). Notably, co-infection with influenza and SARS-CoV-2 has been shown to significantly increase disease severity and risk of death (67). Importantly, one patient in our study who died had a confirmed Influenza A virus coinfection, supporting previous evidence that influenza virus/SARS-CoV-2 co-infections generate a nearly 6-fold increase in fatality risk (68, 69).

Our findings underscore the importance and continuous need for robust monitoring of coinfections and superinfections in critically ill COVID-19 patients. This need remains especially relevant in Latin America, where healthcare systems are still recovering from the prolonged stress of the pandemic and SARS-CoV-2 remains an actively circulating virus. These infections significantly increase patient mortality, hospitalization time, and healthcare costs, not only worsening outcomes, but also contributing to the rising threat of antimicrobial resistance, a critical global health issue further exacerbated during and after the pandemic (70).

Continued investment in microbiological surveillance, rapid diagnostics, and antimicrobial stewardship programs is essential for mitigating the long-term consequences of these infections, particularly in regions with limited healthcare resources.

### 4.1 Limitations

This study provides one of the first detailed descriptions of bacterial and fungal co-infections and superinfections among ICU-hospitalized COVID-19 patients in Ecuador, integrating microbiological, clinical, and temporal data. The use of both culture and molecular diagnostics has allowed for a more comprehensive characterization of pathogens and their onset during hospitalization. However, this study had several limitations that must be acknowledged. First, the small sample size (*n* = 24), retrospective design, and incomplete laboratory data as key biomarkers associated with respiratory disease severity, such as lactate dehydrogenase, D-dimer, platelet count, and troponin level, were not consistently available, limiting the statistical power and generalizability of the findings. Second, the data on symptom duration, disease progression, and comprehensive microbiological testing were incomplete, partly because of delays in hospital diagnostic procedures. Third, Potential biases related to empirical antibiotic use and timing of sampling may also have influenced pathogen detection rates.

## Data Availability

The original contributions presented in this study are included in the article. The raw data supporting the conclusions of this article will be made available by the authors without undue reservation. Further inquiries can be directed to the corresponding author.

## References

[1] Alimohamadi Y Sepandi M Taghdir M Hosamirudsari H. Determine the most common clinical symptoms in COVID-19 patients: A systematic review and meta-analysis. J Prev Med Hyg. (2020) 61:E304–12. doi: 10.15167/2421-4248/jpmh2020.61.3.1530, PMID: 33150219 PMC7595075

[2] Guan W Ni Z Hu Y Liang W Ou C He J . Clinical characteristics of coronavirus disease 2019 in China. N Engl J Med. (2020) 382:1708–20. doi: 10.1056/nejmoa2002032, PMID: 32109013 PMC7092819

[3] Mathieu Edouard Ritchie Hannah Rodés-Guirao Lucas Appel Cameron Gavrilov Daniel Giattino Charlie . (2020) “Mortality risk of COVID-19.” Available online at: https://ourworldindata.org/mortality-risk-covid (Accessed June 27, 2025).

[4] The Lancet. COVID-19 in Latin America—emergency and opportunity. Lancet. (2021) 398:93. doi: 10.1016/S0140-6736(21)01551-8 34246349 PMC8266269

[5] LaRotta J Escobar O Ávila-Aguero ML Torres JP Sini de Almeida R Morales GDC . COVID-19 in Latin America: a snapshot in time and the road ahead. Infect Dis Ther. (2023) 12:389–410. doi: 10.1007/s40121-022-00748-z, PMID: 36633818 PMC9835740

[6] Wu C Chen X Cai Y Xia J Zhou X Xu S . Risk Factors Associated With Acute Respiratory Distress Syndrome and Death in Patients With Coronavirus Disease 2019 Pneumonia in Wuhan, China. JAMA Intern Med. (2019) 180:934–43. doi: 10.1001/jamainternmed.2020.0994, PMID: 32167524 PMC7070509

[7] Feng Y Ling Y Bai T Xie Y Huang J Li J . COVID-19 with different severities: A multicenter study of clinical features. Am J Respir Crit Care Med. (2020) 201:1380–8. doi: 10.1164/rccm.202002-0445OC, PMID: 32275452 PMC7258639

[8] Sharifipour E Shams S Esmkhani M Khodadadi J Fotouhi-Ardakani R Koohpaei A . Evaluation of bacterial co-infections of the respiratory tract in COVID-19 patients admitted to ICU. BMC Infect Dis. (2020) 20:646. doi: 10.1186/s12879-020-05374-z, PMID: 32873235 PMC7461753

[9] Kim D Quinn J Pinsky B Shah NH Brown I. Rates of co-infection between SARS-CoV-2 and other respiratory pathogens. JAMA J Am Med Assoc. (2020) 323:2085–6. doi: 10.1001/jama.2020.6266, PMID: 32293646 PMC7160748

[10] Lai CC Wang CY Hsueh PR. Co-infections among patients with COVID-19: the need for combination therapy with non-anti-SARS-CoV-2 agents? J Microbiol Immunol Infect. (2020) 53:505–12. doi: 10.1016/j.jmii.2020.05.013, PMID: 32482366 PMC7245213

[11] Singh V Upadhyay P Reddy J Granger J. SARS-CoV-2 respiratory co-infections: incidence of viral and bacterial co-pathogens. Int J Infect Dis. (2021) 105:617–20. doi: 10.1016/j.ijid.2021.02.087, PMID: 33640570 PMC7905386

[12] Msemburi W Karlinsky A Knutson V Aleshin-Guendel S Chatterji S Wakefield J. The WHO estimates of excess mortality associated with the COVID-19 pandemic. Nature. (2023) 613:130–7. doi: 10.1038/s41586-022-05522-2, PMID: 36517599 PMC9812776

[13] Secretaría General de Comunicación. (2021) Se registra el primer caso de coronavirus en Ecuador. Available online at: https://www.comunicacion.gob.ec/se-registra-el-primer-caso-de-coronavirus-en-ecuador [Accessed March 2, 2021]

[14] Johns Hopkins Coronavirus Resource Center. (2024) Mortality analyses. Available online at: https://coronavirus.jhu.edu/data/mortality (Accessed January 7, 2025).

[15] Wang L Amin AK Khanna P Aali A McGregor A Bassett P . An observational cohort study of bacterial co-infection and implications for empirical antibiotic therapy in patients presenting with COVID-19 to hospitals in North West London. J Antimicrob Chemother. (2021) 76:796–803. doi: 10.1093/jac/dkaa475, PMID: 33185241 PMC7717240

[16] Musuuza JS Watson L Parmasad V Putman-Buehler N Christensen L Safdar N. Prevalence and outcomes of co-infection and superinfection with SARS-CoV-2 and other pathogens: a systematic review and meta-analysis. PLoS One. (2021) 16:e0251170. doi: 10.1371/journal.pone.0251170, PMID: 33956882 PMC8101968

[17] del Pozo JL. Respiratory co-and superinfections in COVID-19. Rev Esp Quimioter. (2021) 34:69–71. doi: 10.37201/req/s01.20.2021, PMID: 34598432 PMC8683017

[18] Westblade LF Simon MS Satlin MJ. Bacterial coinfections in coronavirus disease 2019. Trends Microbiol. (2021) 29:930–41. doi: 10.1016/j.tim.2021.03.018, PMID: 33934980 PMC8026275

[19] Lansbury L Lim B Baskaran V Lim WS. Co-infections in people with COVID-19: a systematic review and meta-analysis. J Infect. (2020) 81:266–75. doi: 10.1016/j.jinf.2020.05.046, PMID: 32473235 PMC7255350

[20] Swets MC Russell CD Harrison EM Docherty AB Lone N Girvan M . SARS-CoV-2 co-infection with influenza viruses, respiratory syncytial virus, or adenoviruses. Lancet. (2022) 399:1463–4. doi: 10.1016/S0140-6736(22)00383-X, PMID: 35344735 PMC8956294

[21] Nickbakhsh S Mair C Matthews L Reeve R Johnson PCD Thorburn F . Virus-virus interactions impact the population dynamics of influenza and the common cold. Proc Natl Acad Sci USA. (2019) 116:27142–50. doi: 10.1073/pnas.1911083116, PMID: 31843887 PMC6936719

[22] Cowling BJ Fang VJ Nishiura H Chan KH Ng S Ip DKM . Increased risk of noninfluenza respiratory virus infections associated with receipt of inactivated influenza vaccine. Clin Infect Dis. (2012) 54:307. doi: 10.1093/cid/cis307 PMC3404712 22423139

[23] de Oliveira LH Shioda K Valenzuela MT Janusz CB Rearte A Sbarra AN . Declines in pneumonia mortality following the introduction of pneumococcal conjugate vaccines in Latin American and Caribbean countries. Clin Infect Dis. (2021) 73:306–13. doi: 10.1093/cid/ciaa614, PMID: 32448889 PMC8516507

[24] Amin-Chowdhury Z Aiano F Mensah A Sheppard CL Litt D Fry NK . Impact of the coronavirus disease 2019 (COVID-19) pandemic on invasive pneumococcal disease and risk of pneumococcal coinfection with severe acute respiratory syndrome coronavirus 2 (SARS-CoV-2): prospective national cohort study, England. Clin Infect Dis. (2021) 72:e65–75. doi: 10.1093/cid/ciaa1728, PMID: 33196783 PMC7717180

[25] Wang M Wu Q Xu W Qiao B Wang J Zheng H . (2020). Clinical diagnosis of 8274 samples with 2019-novel coronavirus in Wuhan. Available online at: https://www.medrxiv.org/content/10.1101/2020.02.12.20022327v1 (Accessed May 7, 2025).

[26] Antony SJ Almaghlouth NK Heydemann EL. Are coinfections with COVID-19 and influenza low or underreported? An observational study examining current published literature including three new unpublished cases. J Med Virol. (2020) 92:2489–97. doi: 10.1002/jmv.26167, PMID: 32530531

[27] Soto A Quiñones-Laveriano DM Valdivia F Juscamayta-López E Azañero-Haro J Chambi L . Detection of viral and bacterial respiratory pathogens identified by molecular methods in COVID-19 hospitalized patients and its impact on mortality and unfavorable outcomes. Infect Drug Resist. (2021) 14:2795–807. doi: 10.2147/IDR.S306439, PMID: 34321896 PMC8312249

[28] Cataño-Correa JC Cardona-Arias JA Porras Mancilla JP García MT. Bacterial superinfection in adults with COVID-19 hospitalized in two clinics in Medellín-Colombia, 2020. PLoS One. (2021) 16:e0254671. doi: 10.1371/journal.pone.0254671, PMID: 34255801 PMC8277025

[29] Briones Claudett KH Briones-Claudett MH Murillo Vasconez R Benitez Sólis JG Briones Zamora KH Freire AX . Bronchoscopy and molecular diagnostic techniques to identify superimposed infections in COVID-19-associated ARDS: a case series from Ecuador during the second wave. Front Med. (2024) 11:323. doi: 10.3389/fmed.2024.1409323 PMC11557464 39540054

[30] Gómez BJP Pazmiño JPR Quinde GSG Viejó JFG Amaguaña MJM Neira ÉIR . Multidrug-resistant *Klebsiella pneumoniae* in a patient with SARS-Cov-2 pneumonia in an intensive care unit in Guayaquil, Ecuador: a case report. Am J Case Rep. (2022) 23:6498. doi: 10.12659/AJCR.936498 PMC9319302 35864727

[31] Kömeç S Durmuş MA Ceylan AN Korkusuz R. A novel PCR panel for bacterial detection in lower respiratory tract infections: A comparative study with culture results. Pathogens. (2025) 14:1017. doi: 10.3390/pathogens14101017, PMID: 41156628 PMC12567504

[32] Dung TTN Phat VV Vinh C Lan NPH Phuong NLN Ngan LTQ . Development and validation of multiplex real-time PCR for simultaneous detection of six bacterial pathogens causing lower respiratory tract infections and antimicrobial resistance genes. BMC Infect Dis. (2024) 24:164. doi: 10.1186/s12879-024-09028-2, PMID: 38326753 PMC10848345

[33] Rouhi F Erami M Rastgufar S Jahani M Aboutalebian S Soltani S . Quantitative real time PCR for distinction between pneumocystis jirovecii infection/colonization in hospitalized patients. Front Cell Infect Microbiol. (2024) 14:6200. doi: 10.3389/fcimb.2024.1426200, PMID: 39380728 PMC11458531

[34] Bonacorsi S Visseaux B Bouzid D Pareja J Rao SN Manissero D . Systematic review on the correlation of quantitative PCR cycle threshold values of gastrointestinal pathogens with patient clinical presentation and outcomes. Front Med. (2021) 8:1809. doi: 10.3389/fmed.2021.711809, PMID: 34631732 PMC8496934

[35] Diaz JV Appiah J Askie L Baller A Banerjee A Barkley S . COVID-19 Clinical management - Living guidance. Geneva: World Health Organization (2021).

[36] Ferguson ND Fan E Camporota L Antonelli M Anzueto A Beale R . The Berlin definition of ARDS: an expanded rationale, justification, and supplementary material. Intensive Care Med. (2012) 38:1573–82. doi: 10.1007/s00134-012-2682-1, PMID: 22926653

[37] Kalil AC Metersky ML Klompas M Muscedere J Sweeney DA Palmer LB . Management of adults with hospital-acquired and ventilator-associated pneumonia: 2016 clinical practice guidelines by the Infectious Diseases Society of America and the American Thoracic Society. Clin Infect Dis. (2016) 63:e61–e111. doi: 10.1093/cid/ciw353 27418577 PMC4981759

[38] Garcia-Vidal C Sanjuan G Moreno-García E Puerta-Alcalde P Garcia-Pouton N Chumbita M . Incidence of co-infections and superinfections in hospitalized patients with COVID-19: a retrospective cohort study. Clin Microbiol Infect. (2021) 27:83–8. doi: 10.1016/j.cmi.2020.07.041, PMID: 32745596 PMC7836762

[39] Langford BJ So M Raybardhan S Leung V Westwood D MacFadden DR . Bacterial co-infection and secondary infection in patients with COVID-19: a living rapid review and meta-analysis. Clin Microbiol Infect. (2020) 26:1622–9. doi: 10.1016/j.cmi.2020.07.016 32711058 PMC7832079

[40] Chen X Liao B Cheng L Peng X Xu X Li Y . The microbial coinfection in COVID-19. Appl Microbiol Biotechnol. (2020) 104:7777–85. doi: 10.1007/s00253-020-10814-6, PMID: 32780290 PMC7417782

[41] Feldman C Anderson R. The role of co-infections and secondary infections in patients with COVID-19. Pneumonia. (2021) 13:5. doi: 10.1186/s41479-021-00083-w, PMID: 33894790 PMC8068564

[42] Sanghavi SK Bullotta A Husain S Rinaldo CR. Clinical evaluation of multiplex real-time PCR panels for rapid detection of respiratory viral infections. J Med Virol. (2012) 84:162–9. doi: 10.1002/jmv.22186, PMID: 22052551 PMC7166524

[43] Freire-Paspuel B Garcia-Bereguiain MA. Analytical sensitivity and clinical performance of a triplex RT-qPCR assay using CDC N1, N2, and RP targets for SARS-CoV-2 diagnosis. Int J Infect Dis. (2021) 102:14–6. doi: 10.1016/j.ijid.2020.10.047, PMID: 33115681 PMC7585718

[44] Freire-Paspuel B Vega-Mariño P Velez A Cruz M Garcia-Bereguiain MA. Sample pooling of RNA extracts to speed up SARS-CoV-2 diagnosis using CDC FDA EUA RT-qPCR kit. Virus Res. (2020) 290:198173. doi: 10.1016/j.virusres.2020.198173, PMID: 32979475 PMC7513759

[45] Freire-Paspuel B Vega-Mariño P Velez A Cruz M Perez F Garcia-Bereguiain MA. Analytical and clinical comparison of Viasure (CerTest biotec) and 2019-nCoV CDC (IDT) RT-qPCR kits for SARS-CoV2 diagnosis. Virology. (2021) 553:154–6. doi: 10.1016/j.virol.2020.10.010, PMID: 33278737 PMC7673214

[46] Carvalho MDGS Tondella ML McCaustland K Weidlich L McGee L Mayer LW . Evaluation and improvement of real-time PCR assays targeting *lytA*, *ply*, and *psaA* genes for detection of pneumococcal DNA. J Clin Microbiol. (2007) 45:2460–6. doi: 10.1128/JCM.02498-06 17537936 PMC1951257

[47] Centers for disease control and prevention. (2014). Streptococcus lab resources and protocols. Available online at: https://www.cdc.gov/streplab/pneumococcus/resources.html [Accessed June 10, 2023]

[48] Morales-Jadán D Muslin C Viteri-Dávila C Coronel B Castro-Rodríguez B Vallejo-Janeta AP . Coinfection of SARS-CoV-2 with other respiratory pathogens in outpatients from Ecuador. Front Public Health. (2023) 11:4632. doi: 10.3389/fpubh.2023.1264632, PMID: 37965509 PMC10641819

[49] Li Z Chen Z Chen L Zhan Y Li S Cheng J . Coinfection with SARS-CoV-2 and other respiratory pathogens in patients with COVID-19 in Guangzhou, China. J Med Virol. (2020) 92:2381–3. doi: 10.1002/jmv.26073, PMID: 32462695 PMC7283743

[50] Bhatraju PK Ghassemieh BJ Nichols M Kim R Jerome KR Nalla AK . Covid-19 in critically ill patients in the Seattle region — case series. N Engl J Med. (2020) 382:2012–22. doi: 10.1056/NEJMoa2004500, PMID: 32227758 PMC7143164

[51] Contou D Claudinon A Pajot O Micaëlo M Longuet Flandre P Dubert M . Bacterial and viral co-infections in patients with severe SARS-CoV-2 pneumonia admitted to a French ICU. Ann Intensive Care. (2020) 10:119. doi: 10.1186/s13613-020-00736-x, PMID: 32894364 PMC7475952

[52] Silva DL Lima CM Magalhães VCR Baltazar LM Peres NTA Caligiorne RB . Fungal and bacterial coinfections increase mortality of severely ill COVID-19 patients. J Hosp Infect. (2021) 113:145–54. doi: 10.1016/j.jhin.2021.04.001, PMID: 33852950 PMC8056850

[53] Santos AP Gonçalves LC Oliveira ACC Queiroz PHP Ito CRM Santos MO . Bacterial co-infection in patients with COVID-19 hospitalized (ICU and not ICU): review and meta-analysis. Antibiotics. (2022) 11:894. doi: 10.3390/antibiotics11070894, PMID: 35884147 PMC9312179

[54] Giacobbe DR Battaglini D Ball L Brunetti I Bruzzone B Codda G . Bloodstream infections in critically ill patients with COVID-19. Eur J Clin Investig. (2020) 50:e13319. doi: 10.1111/eci.13319, PMID: 32535894 PMC7323143

[55] Rosenthal VD Myatra SN Divatia JV Biswas S Shrivastava A Al-Ruzzieh MA . The impact of COVID-19 on health care–associated infections in intensive care units in low- and middle-income countries: international nosocomial infection control consortium (INICC) findings. Int J Infect Dis. (2022) 118:83–8. doi: 10.1016/j.ijid.2022.02.041, PMID: 35218928 PMC8866162

[56] Rosenthal VD Duszynska W Ider B-E Gurskis V Al-Ruzzieh MA Myatra SN . International nosocomial infection control consortium (INICC) report, data summary of 45 countries for 2013-2018, adult and Pediatric units, Device-associated module. Am J Infect Control. (2021) 49:1267–74. doi: 10.1016/j.ajic.2021.04.077, PMID: 33901588

[57] Satta G Rawson TM Moore LSP. Coronavirus disease 2019 (COVID-19) impact on central-line-associated bloodstream infections (CLABSI): a systematic review. Infect Prev Pract. (2023) 5:313. doi: 10.1016/j.infpip.2023.100313, PMID: 37920796 PMC10618700

[58] Najafi-Olya Z Heydarifard Z Looha MA Ahmadi AS Yarhamadi N Safaei M. Antibiotic resistance and bacterial co-infections in COVID-19 patients in Iran: a systematic review and meta-analysis of hospitalized and non-hospitalized cases. BMC Infect Dis. (2025) 25:1197. doi: 10.1186/s12879-025-11643-6, PMID: 41023991 PMC12482416

[59] Calvo M Stefani S Migliorisi G. Bacterial infections in intensive care units: epidemiological and microbiological aspects. Antibiotics. (2024) 13:238. doi: 10.3390/antibiotics13030238, PMID: 38534673 PMC10967584

[60] Jiang S Liu P Xiong G Yang Z Wang M Li Y . Coinfection of SARS-CoV-2 and multiple respiratory pathogens in children. Clin Chem Lab Med. (2020) 58:1160–1. doi: 10.1515/cclm-2020-0434, PMID: 32301747

[61] Dikranian L Barry S Ata A Chiotos K Gist K Bhalala U . SARS-CoV-2 with concurrent respiratory viral infection as a risk factor for a higher level of Care in Hospitalized Pediatric Patients. Pediatr Emerg Care. (2022) 38:472–6. doi: 10.1097/PEC.0000000000002814, PMID: 36040468 PMC9426307

[62] Dueñas D Daza J Liscano Y. Coinfections and superinfections associated with COVID-19 in Colombia: a narrative review. Medicina. (2023) 59:1336. doi: 10.3390/medicina59071336, PMID: 37512147 PMC10385172

[63] Toro-Rendon LG Rojas-Gualdron DF Rodriguez-Tobon FA Perez-Urrego CA Palacios-Barahona U. Direct costs of hospital care according to coinfection in adult COVID-19 patients. Infectio. (2023) 27:71–7. doi: 10.22354/24223794.1125

[64] Rawson TM Moore LSP Zhu N Ranganathan N Skolimowska K Gilchrist M . Bacterial and fungal coinfection in individuals with coronavirus: A rapid review to support COVID-19 antimicrobial prescribing. Clin Infect Dis. (2020) 71:2459–68. doi: 10.1093/cid/ciaa530, PMID: 32358954 PMC7197596

[65] Siegel SJ Roche AM Weiser JN. Influenza promotes pneumococcal growth during coinfection by providing host Sialylated substrates as a nutrient source. Cell Host Microbe. (2014) 16:55–67. doi: 10.1016/j.chom.2014.06.005, PMID: 25011108 PMC4096718

[66] Jochems SP Marcon F Carniel BF Holloway M Mitsi E Smith E . Inflammation induced by influenza virus impairs human innate immune control of pneumococcus. Nat Immunol. (2018) 19:1299–308. doi: 10.1038/s41590-018-0231-y, PMID: 30374129 PMC6241853

[67] Stowe J Tessier E Zhao H Guy R Muller-Pebody B Zambon M . Interactions between SARS-CoV-2 and influenza, and the impact of coinfection on disease severity: a test-negative design. Int J Epidemiol. (2021) 50:1124–33. doi: 10.1093/ije/dyab081, PMID: 33942104 PMC8135706

[68] Dadashi M Khaleghnejad S Abedi Elkhichi P Goudarzi M Goudarzi H Taghavi A . COVID-19 and influenza co-infection: A systematic review and Meta-analysis. Front Med. (2021) 8:1469. doi: 10.3389/fmed.2021.681469, PMID: 34249971 PMC8267808

[69] Pinky L Dobrovolny HM. SARS-CoV-2 coinfections: could influenza and the common cold be beneficial? J Med Virol. (2020) 92:2623–30. doi: 10.1002/jmv.26098, PMID: 32557776 PMC7300957

[70] Rasizadeh R Milani ES Aghbash PS Arefi V Fathi H Nahand JS . Beyond the virus: exploring coinfections in the COVID-19 pandemic. Open Microbiol J. (2023) 17:202. doi: 10.2174/0118742858274177231110050202

